# Endolysins: targeting *Streptococcus pneumoniae* in its major anatomical niches of infection and addressing the hurdles

**DOI:** 10.3389/fmicb.2025.1660791

**Published:** 2025-10-03

**Authors:** Giovanna Castallanos, Norberto Gonzalez-Juarbe, Daniel C. Nelson

**Affiliations:** ^1^Institute for Bioscience and Biotechnology Research, University of Maryland, Rockville, MD, United States; ^2^Department of Cell Biology and Molecular Genetics, University of Maryland, College Park, MD, United States; ^3^Department of Veterinary Medicine, University of Maryland, College Park, MD, United States

**Keywords:** endolysin, peptidoglycan hydrolase, bacteriophage, *Streptococcus pneumoniae*, pneumonia, otitis media, meningitis, sepsis

## Abstract

*Streptococcus pneumoniae* is responsible for causing a range of diseases, from self-limiting otitis media to more severe disease such as pneumonia, sepsis, and meningitis. Vaccines and antibiotics have successfully decreased this disease burden; however, serotype replacement due to vaccines and antibiotic resistance remain issues pointing to the need for further research into alternative methods of treatment. *S. pneumoniae* continues to pose a global threat to young children and the elderly. Endolysins, which function as peptidoglycan hydrolases, are an attractive alternative treatment given their rapid bactericidal activity, specificity, and lack of noted resistance. This review considers the uses of endolysins within the main niches of infection for *S. pneumoniae*: pulmonary, middle ear, and the bloodstream and central nervous system. Therapeutic hurdles, such as delivery and mechanical barrier challenges, are discussed and endolysin-focused solutions to circumvent these challenges are proposed. The ability to address niche-specific hurdles using endolysins will allow for an increase in effective therapies against *S. pneumoniae.*

## 1 Introduction

**Figure 1:**
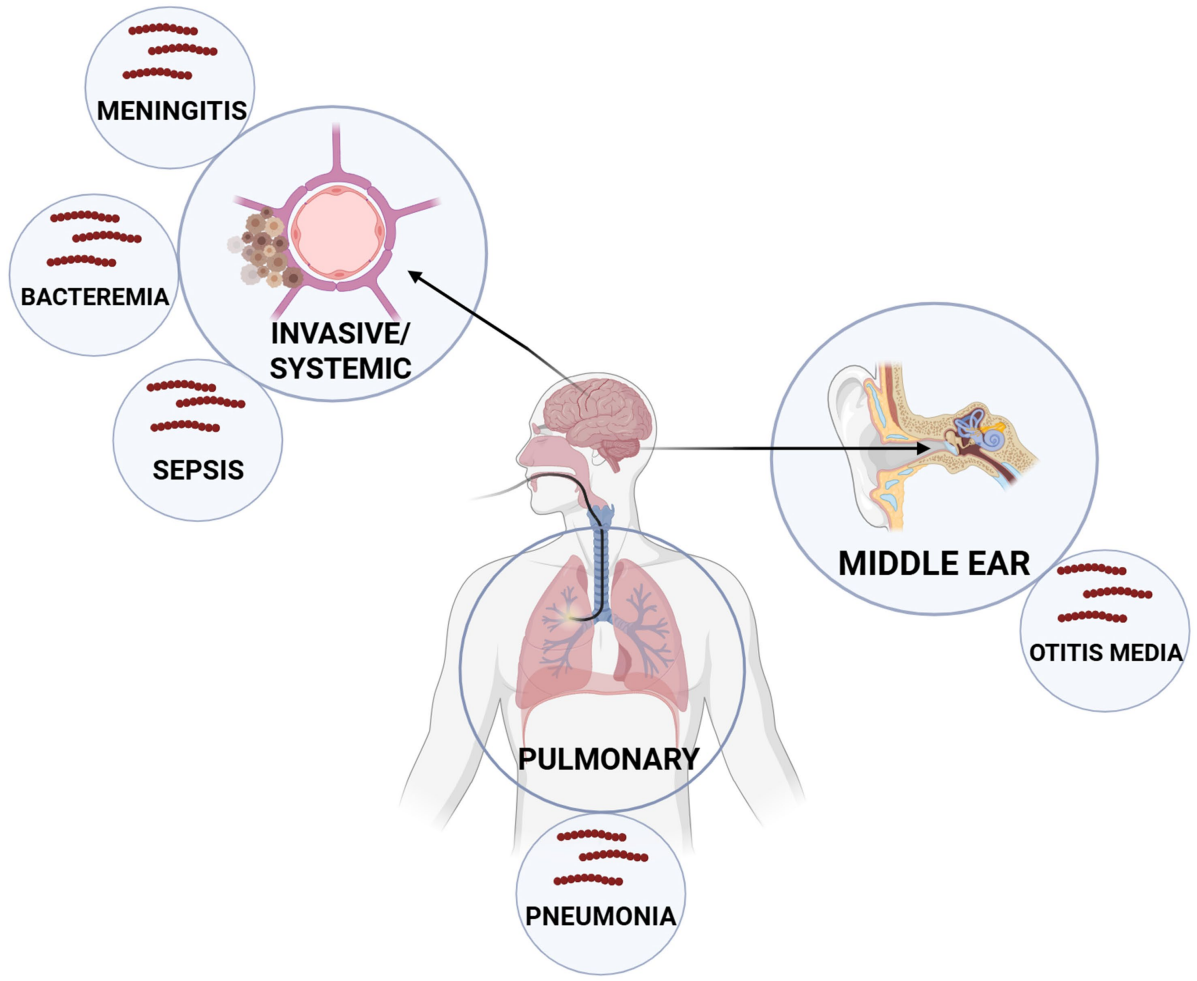
Major anatomical niches and associated diseases caused by *Streptococcus pneumoniae*. *S. pneumoniae* colonizes the nasopharynx, where it migrates, primarily infecting the pulmonary system (pneumonia) or the middle ear (otitis media). However, it can also disseminate systemically to cause invasive diseases such as sepsis, bacteremia, and meningitis. These distinct infection sites represent key targets for therapeutic intervention and highlight the diverse clinical manifestations of pneumococcal disease.

*Streptococcus pneumoniae* is a Gram-positive bacterium known for colonizing and infecting the mucosal membranes of the upper respiratory tract, particularly in elderly individuals and children under 5 years of age. Although both adults and children are often asymptomatic carriers, an increased bacterial load can lead to diseases such as pneumonia, otitis media, sepsis, and meningitis (Austrian, 1981; Shenoy and Orihuela, 2016; Weiser et al., 2018) (Figure 1). The Active Bacterial Core Surveillance system reported 3,297 cases of *S. pneumoniae* infection and 361 deaths per 100,000 people in the United States in 2018 (CDC, 2018). Additionally, the Centers for Disease Control and Prevention has identified *S. pneumoniae* as a top-priority threat pathogen due to its high disease burden, antibiotic resistance, serotype replacement, vaccine escape, transmission, and asymptomatic carriage (CDC, 2019).

The disease mechanism requires shedding of the bacterium from mucosal membranes followed by either direct contact or airborne transmission to an uninfected person. This is facilitated by the ability of *S. pneumoniae* to release of pneumolysin, a pore-forming toxin, which causes host cell destruction, epithelial and endothelial barrier disruption, immune response modulation, and inflammation (Weiser et al., 2018). Viral co-infections, like influenza A, enhance the distribution of *S. pneumoniae* by increasing inflammation and triggering mucosal shedding. To protect itself from the immune system, *S. pneumoniae* uses multiple evasion strategies such as production of a polysaccharide capsule that inhibits phagocytosis, display of surface proteins that block complement activation, and the aforementioned pneumolysin-mediated cytotoxicity. *S. pneumoniae* also develops biofilms on mucosal surfaces that allow it to persist on biotic surfaces and leads to repeated chronic infections. The treatment of pneumococcal diseases relies on broad-spectrum antibiotics and preventative vaccines, yet their long-term effectiveness and sustainability remain limited (Kato, 2024).

Vaccines are primarily designed to target the polysaccharide capsule of *S. pneumoniae*, but only a limited number of capsule serotypes are represented across the two main types of vaccines: polysaccharide vaccines and polysaccharide-conjugate vaccines (PCVs) (Table 1) (Owusu-Edusei et al., 2022). PCVs combine polysaccharide antigens with diphtheria-derived cross-reactive protein domains and an aluminum phosphate adjuvant to create conjugates that boost immunogenicity while generating stronger immune responses (Davies et al., 2022). The introduction of PCVs resulted in a 51% reduction in child mortality between 2000 and 2015 (Wahl et al., 2018). Nonetheless, vaccine efficacy is limited to only the serotypes included in the formulation: Polysaccharide vaccines include up to 24 serotypes, whereas currently approved conjugate vaccines protect against 13, 15, 20, or 21 serotypes depending on the vaccine, although more than 100 capsular serotypes have been identified for *S. pneumoniae* (CDC, 2010; Haber et al., 2016; Keller et al., 2016). The restricted coverage of serotypes by current vaccines can result in serotype replacement, whereby *S. pneumoniae* with non-vaccine capsule serotypes can become the main source of infection (Lo et al., 2019). Toward this end, Capvaxie (PCV21), which as approved by the FDA in July, 2024, excludes 11 serotypes that are present in prior PCV vaccines and includes 8 new serotypes not found in any previous PCV vaccines (Kobayashi et al., 2024). Furthermore, vaccine effectiveness may be reduced in carrier populations due to age-associated immune function. Children have the highest rates of *S. pneumoniae* carriage, ranging from 27 to 65%, whereas adults exhibit carriage rates of less than 10% (Westerink et al., 2012; Castelo-Branco and Soveral, 2014; Albright et al., 2016). Additionally, elderly individuals who received PCV vaccines targeting serotypes 14 and 23F exhibited no increases in IgM levels and had lower opsonophagocytic activity compared to younger adults (Leggat et al., 2013). Likewise, older individuals had lower IgG antibody titers against common *S. pneumoniae* serotypes (Simell et al., 2008). These findings suggest that the most frequent carriers of *S. pneumoniae*, i.e., children and elderly individuals, may remain susceptible to infection despite vaccination efforts. Compounding this issue, vaccine uptake remains suboptimal: in 2018, only 23% of adults aged 19–64 and 69% of adults over 65 years of age in the United States were vaccinated (Owusu-Edusei et al., 2022).

**Table 1:** Serotype coverage of currently recommended pneumococcal vaccines.

Vaccine (brand) type	Serotypes covered
PCV13 (Prevnar 13) Conjugate	1, 3, 4, 5, 6A, 6B, 7F, 9 V, 14, 18C, 19A, 19F, 23F
PCV15 (Vaxneuvance) Conjugate	All PCV13 serotypes plus 22F, 33F
PCV20 (Prevnar 20) Conjugate	All PCV15 serotypes plus 8, 10A, 11A, 12F, 15B
PCV21^*^ (Capvaxive) Conjugate	3, 6A, 7F, 8, 9 N, 10A, 11A, 12F, 15A, 15B, 15C, 16F, 17F, 19A, 20A, 22F, 23A, 23B, 24F, 31, 33F, 35B
PPSV23 (Pneumovax 23) Polysaccharide	1, 2, 3, 4, 5, 6B, 7F, 8, 9 N, 9 V, 10A, 11A, 12F, 14, 15B, 17F, 18C, 19A, 19F, 20, 22F, 23F, 33F

* Although PCV21 is a 21-valent vaccine, 22 serovars are recognized since the de-O-acetylated 15B antigen cross-reacts with 15C.

The ongoing challenge of suboptimal vaccine protection combined with rising antibiotic resistance creates a parallel threat. Serotype replacement has contributed to a steady rise in antibiotic resistance among replacement serovars (Lo et al., 2019). *S. pneumoniae* has demonstrated resistance to three major classes of antibiotics: Resistance to *β*-lactams occurs through modifications of penicillin-binding proteins; resistance to macrolides is due to *ermB*, which alters the ribosomal subunit structure; and fluoroquinolones encounter resistance through mutations in the quinolone resistance-determining regions of DNA gyrase and topoisomerase IV genes (Cilloniz et al., 2018). While vaccines have reduced the overall mortality associated with *S. pneumoniae* infection, the emergence of serotype replacement and antibiotic resistance reveal a need for further development of alternative treatment strategies.

### 1.1 Endolysin as an advantageous treatment for *S. pneumoniae*

Bacteriophages (or phages) are viruses that selectively infect and lyse bacteria and have re-emerged as promising alternatives to antibiotics. While phages offer specificity and the potential for self-amplification within bacterial hosts, their therapeutic use remains constrained by several limitations. These include rapid clearance by the host immune system, potential emergence of bacterial resistance via receptor modification or Clustered Regularly Interspaced Short Palindromic Repeats–CRISPR-associated systems, and the lack of standardized protocols for clinical-grade manufacturing and regulatory approval, all of which complicate clinical adoption (Skurnik et al., 2025).

To address these issues, attention has increasingly turned to a class of phage-derived enzymes, particularly a group of peptidoglycan hydrolases known as endolysins. These enzymes are produced during the lytic phase of the phage life cycle and act from the inside-out to release progeny phage, accessing the peptidoglycan only after phage produced holins permeabilize the bacterial membrane (Oliveira et al., 2018). However, when applied externally to Gram-positive bacteria as purified proteins, endolysins act from the outside-in, a phenomenon known as “lysis from without.” In this case, the peptidoglycan substrate is identical, but the enzyme has direct access. Once the peptidoglycan is sufficiently degraded, the underlying membrane ruptures from osmotic lysis, resulting in bacterial cell death. This outside-in activity allows endolysins to be harnessed for therapeutic use, all while retaining specificity for the peptidoglycan of the original phage host species (Rahman et al., 2021).

**Figure 2:**
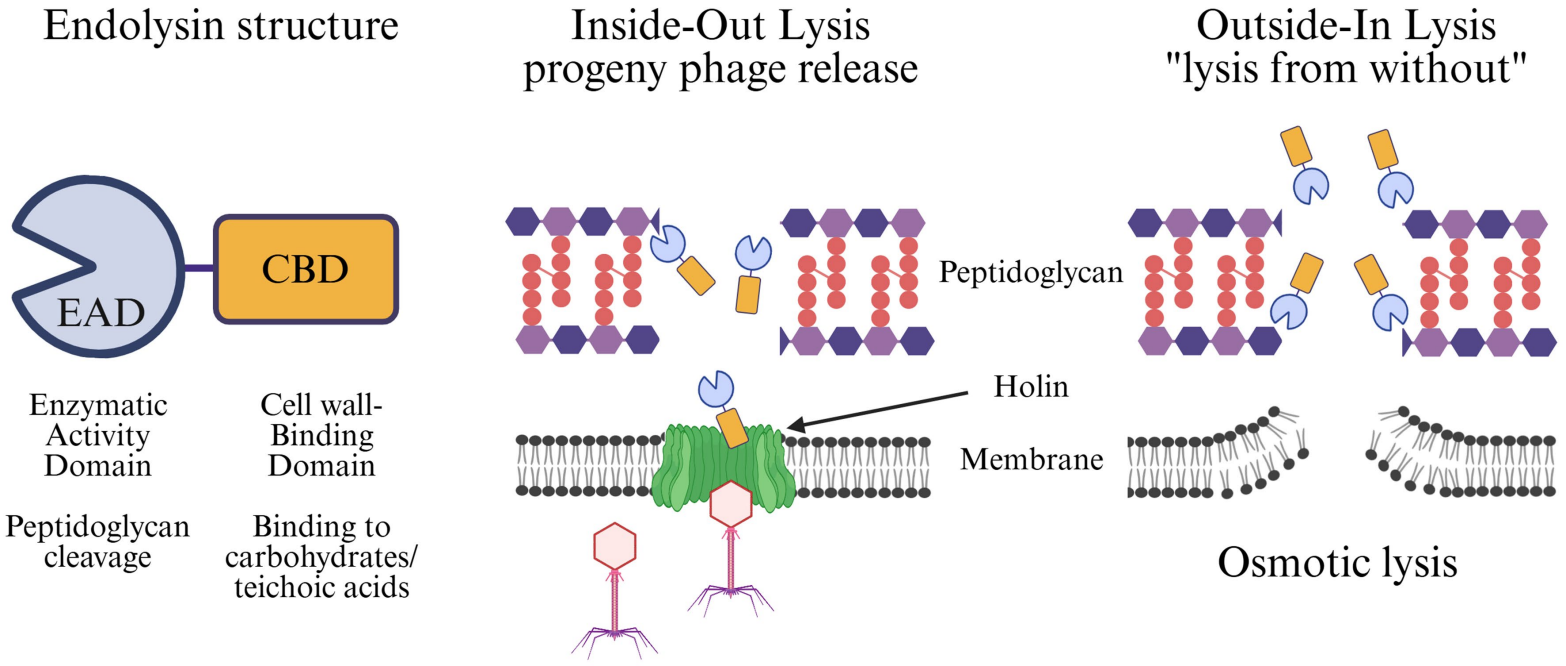
Structure and mechanisms of endolysin-mediated bacterial lysis. Endolysins are modular proteins composed of an N-terminal Enzymatic Activity Domain (EAD), which cleaves specific bonds in the peptidoglycan, and a C-terminal Cell wall-Binding Domain (CBD), which mediates recognition of cell wall carbohydrates or teichoic acids (left). Endolysins can act via two distinct modes: (middle) *Inside-out lysis* during the phage lytic cycle, where holins form pores in the bacterial membrane, allowing endolysins to degrade the peptidoglycan and release progeny phage, and (right) *Outside-in lysis*, also knowns as “lysis from without,” where externally applied endolysins directly access and cleave peptidoglycan, weakening the cell wall and leading to osmotic lysis of the bacterium.

Endolysins derived from phage that infect Gram-positive hosts have evolved a modular structure. Typically, they are composed of an N-terminal enzymatically active domain (EAD) and a C-terminal cell wall binding domain (CBD) (Fischetti, 2010; Roach and Donovan, 2015; Ajuebor et al., 2016; Rahman et al., 2021). The modular architecture of pneumococcal endolysins and their mechanisms of lysis are illustrated in Figure 2. Endolysin EADs can be classified into five enzymatic categories based on the specific bonds they cleave within the bacterial peptidoglycan: acetylmuramidases, transglycosylases, glucosaminidases, amidases, and endopeptidases (Abdelrahman et al., 2021). In contrast, the CBD confers specificity by recognizing conserved epitopes, often carbohydrates or teichoic acids, on the bacterial cell wall that facilitate targeted binding. Depending on the epitope, the recognition of the CBD can be to the genus, species, sub-species, or even strain level. In the case of pneumococcal-specific endolysins, the CBDs typically recognize phosphorylcholine in the wall teichoic acids, a signature unique to *S. pneumoniae*. Upon binding to the bacterial surface, the EAD cleaves its designated bond, resulting in eventual osmotic cell lysis when enough bonds have been cleaved to destabilize the peptidoglycan superstructure.

Extensive *in vitro* and *in vivo* studies have shown that endolysins maintain potent lytic activity when applied to susceptible bacteria (Schmelcher and Loessner, 2021). Most notably, endolysins are unaffected by many resistance mechanisms common to traditional antibiotics, including efflux pumps, altered penicillin-binding proteins, and modifications of metabolic pathways (Nelson et al., 2012). Their high specificity, rapid bactericidal activity, and low rates of resistance development make endolysins attractive candidates for therapeutic use.

### 1.2 Known *S. pneumoniae* endolysins

The endolysins Cpl-1, Cpl-7, and Cpl-9 were among the first enzymes studied to exhibit the modular EAD/CBD architecture (Garcia et al., 1990). More recently, *in vivo* efficacy studies have shown effectiveness for several *S. pneumoniae* endolysins (Table 2). Pal, an endolysin derived from the Dp-1 phage, was the first to be evaluated for *in vivo* efficacy, demonstrating a protective effect in a mouse nasopharyngeal colonization model (Loeffler et al., 2001). However, Cpl-1 is the most extensively studied *S. pneumoniae* endolysin, exhibiting efficacy across multiple *in vivo* models, including intravenous bacteremia (Loeffler et al., 2003), mouse otitis media (McCullers et al., 2007), severe pneumonia (Witzenrath et al., 2009; Doehn et al., 2013), rat endocarditis (Entenza et al., 2005), and pneumococcal meningitis when administered intracisternally (Grandgirard et al., 2008). Recently, Alreja et al. (2024) identified a *S. pneumoniae* endolysin, SP-CHAP, which contains a cysteine, histidine-dependent amidohydrolase/peptidase (CHAP) domain within its EAD. SP-CHAP demonstrated superior activity compared to Cpl-1 across all major *S. pneumoniae* serotypes tested and was effective in eradicating pneumococcal biofilms at concentrations as low as 1.56 μg/mL.

**Table 2:** Endolysins bacteriolytic against *Streptococcus pneumoniae* and their respective animal models.

Endolysin(s)	Type	Model	Route/Results
Pal	Wild-type	Mouse nasopharyngeal colonization (Loeffler et al., 2001)	Nasal: Rapid dose–response killing of *S. pneumoniae* prevented colonization
Cpl-1	Wild-type	Mouse bacteremia (Loeffler et al., 2003) Mouse nasopharyngeal colonization (Loeffler et al., 2003) Mouse pneumonia (Witzenrath et al., 2009; Doehn et al., 2013) Rat endocarditis (Entenza et al., 2005) Mouse otitis media (McCullers et al., 2007) Rat meningitis (Grandgirard et al., 2008)	Intravenous: At a dose of 2,000 μg/mouse, 100% of mice survived compared to 20% survival in control group Intranasal: No evidence of *S. pneumoniae* in nasal wash after treatment with 1,000 μg Cpl-1 Intraperitoneal: Treatment with 1,000 μg Cpl-1 24 h post-infection resulted in 100% survival in mice with fatal pneumonia. Inhalation: In the second study, administration of 400 μg of aerosolized Cpl-1 reduced mortality by 80% Intravenous: A high, continuous dose (250 mg/kg) resulted in no detectable *S. pneumoniae* in blood 30 min post-treatment Intranasal: Mice colonized with *S. pneumoniae* and treated with 1,000 μg Cpl-1 did not develop acute otitis media Intracisternal: A single injection of Cpl-1 (20 mg/kg) resulted in rapid clearance of *S. pneumoniae*
Pal/Cpl-1	Wild-type	Mouse sepsis (Jado et al., 2003)	Intraperitoneal: Synergistic effects observed with a combination of 2.5 μg each of Cpl-1 and Pal in the treatment of murine sepsis
SP-CHAP	Wild-type	Mouse nasopharyngeal colonization (Alreja et al., 2024)	Intranasal: Treatment with 60 μg led to a reduction in *S. pneumoniae* colonization, with greater efficacy compared to Cpl-1
Cpl-711	Engineered chimera of Cpl-1 and Cpl-7S	Mouse bacteremia (Díez-Martínez et al., 2015)	Intraperitoneal: Enzyme provided dose–response protection (25–500 μg) in a fatal bacteremia model as well as a reduction in biofilm formation
ClyJ	Engineered chimera of PlyCA and gp20	Mouse bacteremia (Yang et al., 2019)	Intraperitoneal: Endolysin showed dose–response, protecting 100% at a dose of 400 μg/mouse
ClyJ-3	Linker-engineered version of ClyJ	Mouse bacteremia (Yang et al., 2020)	Intraperitoneal: Endolysin showed dose–response, protecting 100% of mice at a dose of 100 μg/mouse
ClyJ-3 m	Engineered truncation of ClyJ-3, resulting in a monomer	Mouse bacteremia (Luo et al., 2020)	Intraperitoneal: Enzyme showed a dose response, protecting 70% of mice at a dose of 2.32 μug/mouse

Several other *S. pneumoniae* endolysins have been developed through chimeragenesis and protein engineering. One early example involved the construction of chimeric proteins by switching the EAD and CBD domains of Cpl-1 and LytA, and autolysin, which preserved enzymatic activity and choline-binding affinity common to *S. pneumoniae* endolysin CBDs (Diaz et al., 1990). Additional engineered variants include Cpl-7S, derived from Cpl-7 by inverting the charge of 15 amino acids within its CBD (Diez-Martinez et al., 2013). A further fusion of domains derived from Cpl-7S and Cpl-1 resulted in creation of Cpl-711, which demonstrated enhanced bactericidal activity both *in vitro* and *in vivo* when compared to the parental enzymes (Díez-Martínez et al., 2015). Similar, iterative rounds of engineering were also used to create ClyJ (Yang et al., 2019), its variant, ClyJ-3, featuring a shortened linker region between EAD and CBD (Yang et al., 2020), and its monomeric mutant, ClyJ-3 m (Luo et al., 2020). Each variant displayed greater antimicrobial activity than its predecessor.

The strategic combination of endolysins with EADs that target different bonds in the peptidoglycan matrix has also been shown to produce synergistic effects. These combinations can enhance antibacterial efficacy, reduce the required therapeutic dose, and mitigate the risk of resistance. For example, a synergistic interaction between Pal and Cpl-1 has been observed *in vivo* (Jado et al., 2003). Pal, with an EAD characterized as an *N*-acetylmuramoyl-*L*-alanine amidase, cleaves between the glycan strand and the stem peptide of the peptidoglycan, whereas Cpl-1 functions as an *N*-acetylmuramidase, cleaving directly within the glycan backbone. When administered at 2.5 μg each, the Pal/Cpl-1 combination conferred superior protection in mice challenged intraperitoneally with a lethal dose of *S. pneumoniae* compared to a 200 μg dose of either enzyme alone (Jado et al., 2003).

### 1.3 Site-targeted treatment for pneumococcal disease

The advantages of endolysins over conventional vaccines and antibiotics, and the availability to engineer and purify *S. pneumoniae* endolysins, make these agents promising candidates for development of new therapeutics. To date, however, only endolysins targeting *Staphylococcus aureus* and *Clostridioides difficile* have advanced to human clinical trials. These include exebacase (also known as CF-301), LSVT-1701 (formerly SAL200), and XZ.700 (Jun et al., 2017; Fowler et al., 2020; Kuiper et al., 2021), all of which target *S. aureus*, and LMN-201, which targets *C. difficile*. Notably, LSVT-1701 was granted fast-track designation by the U.S. Food and Drug Administration based on its efficacy against over 400 clinical isolates of *S. aureus* (Wire et al., 2022). As preclinical research continues to expand on endolysins, those targeting a wider range of pathogens, such as *S. pneumoniae*, are likely to progress toward clinical development.

**Figure 3:**
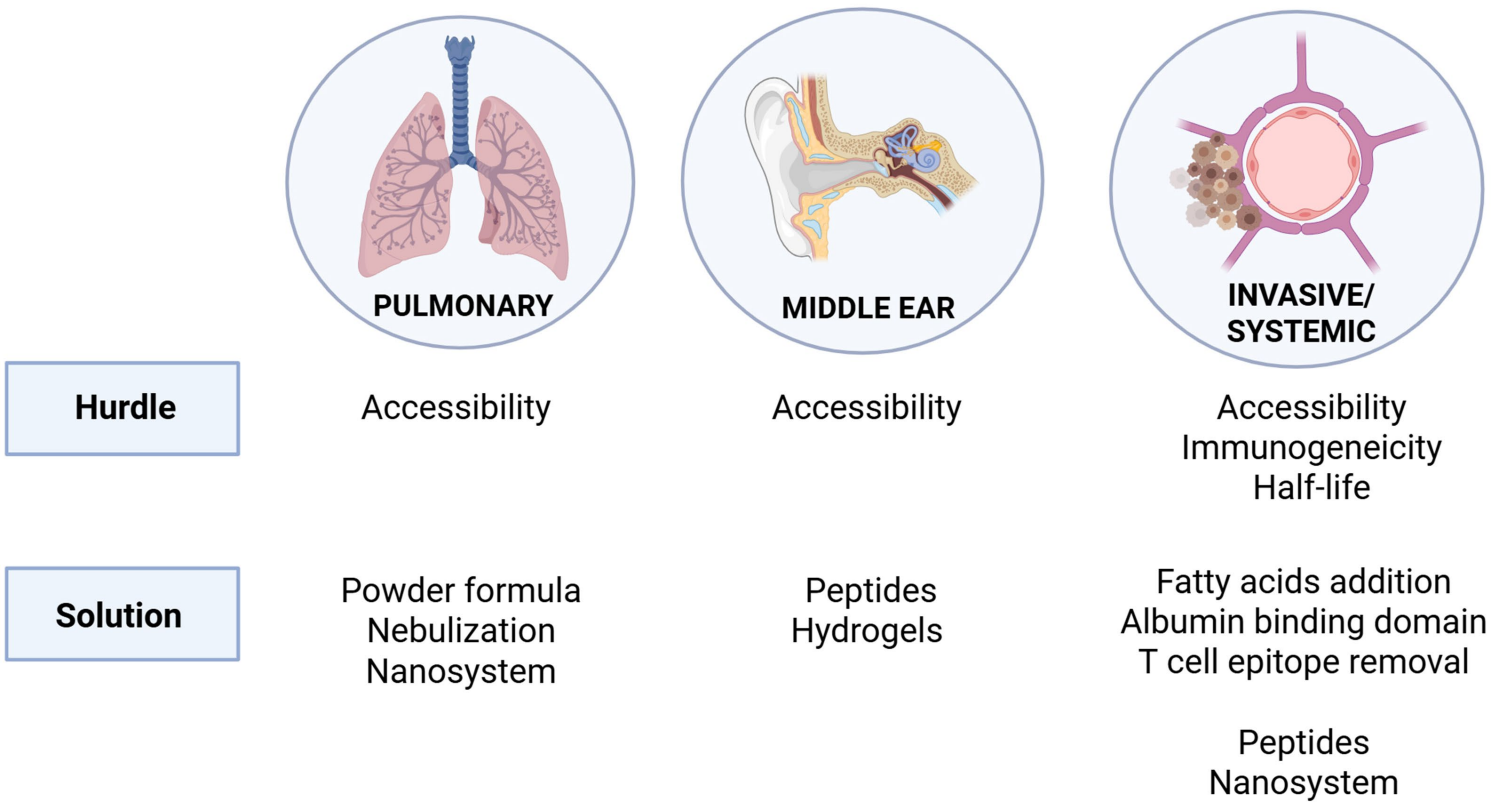
Therapeutic hurdles and potential solutions for targeting *S. pneumoniae* in its major niches of infection. The pulmonary and middle ear environments pose accessibility challenges for endolysin delivery, addressed through strategies such as powder formulations, nebulization, nanosystems, peptides, and hydrogels. In invasive/systemic infections, additional hurdles include immunogenicity and limited half-life. Proposed solutions include fatty acid conjugation, incorporation of albumin-binding domains, removal of T cell epitopes, as well as peptide-based and nanosystem delivery approaches.

Defining the anatomical site of infection to be targeted is essential for advancing pneumococcal endolysins into clinical use. Figure 3 provides an overview of therapeutic hurdles and potential solutions across all major niches. These will be described in greater detail in the following sections. All pneumococcal diseases begin with asymptomatic colonization of the upper respiratory tract mucosa, which is a necessary precursor for transmission to other tissues and anatomical sites (Bogaert et al., 2004). The principal anatomical niches affected by *S. pneumoniae* are: (1) the respiratory tract, where it causes pneumonia and other pulmonary diseases; (2) the middle ear, which results in otitis media; and (3) the bloodstream and central nervous system, which leads to bacteremia, sepsis, and meningitis after crossing the blood–brain barrier. Therapeutic intervention becomes complicated because each anatomical niche has its own distinct physiological and immunological barriers. This review focuses on identifying the unique treatment challenges of *S. pneumoniae* infection within different bodily niches and emphasizes ongoing research and potential future strategies to enhance endolysin effectiveness against pneumococcal diseases.

## 2 Anatomical niche: pulmonary infection hurdles and solutions

Respiratory tract infections remain one of the most common causes of death in third world countries, with symptoms including cough, fever, shortness of breath, pain when breathing, and nausea (Ashrafi-Asgarabad et al., 2023). Upon colonization of the nasopharynx, changes in the host immune response or co-infection with a viral agent, such as influenza or respiratory syncytial virus, can enhance *S. pneumoniae* migration to the lungs. The expression of phosphorylcholine on the *S. pneumoniae* surface facilitates bacterial adherence to platelet-activating factor, while choline-binding protein A binds to human secretory components (e.g., epithelial glycoprotein, polymeric immunoglobulin receptor, etc.) on the host, contributing to the adherence and colonization of *S. pneumoniae* (Kadioglu et al., 2008). Once in the lungs, *S. pneumoniae* adheres to the epithelial cells through surface proteins, activating inflammatory factors. These, in turn, upregulate host receptors that enable internalization of *S. pneumoniae* in the epithelial cells leading to invasion and pneumonia disease (Bogaert et al., 2004). Community-acquired pneumonia is the most common form of pneumonia where *S. pneumoniae* is spread via personal contact with aerosol droplets within the community (Brooks and Mias, 2018).

Aside from the previously mentioned problems with the current treatments of antibiotics and vaccines, another important hurdle for treatment of pulmonary infections is that of the delivery method. Oral or intravenous injections have been proven suboptimal given the indirect route. Such indirect approaches result in lower drug concentrations in the lungs due to low half-life, measured *in vivo* at ~20 min for the endolysin Cpl-1 (Loeffler et al., 2003). Increasing drug concentrations to deliver adequate enzyme to the lungs raises concerns regarding toxicity. This underscores the need to further investigate methods for direct lung delivery of endolysins through aerosolization (Doehn et al., 2013), nebulization (Wang et al., 2020), powders (Wang et al., 2023), and nanotechnology (Falciani et al., 2020).

### 2.1 Delivery methods explored

#### 2.1.1 Aerosolization

Doehn et al. (2013) showed that Cpl-1 could be delivered via aerosol inhalation 24 h post-lethal *S. pneumoniae* infection to successfully rescue mice. Upon infection, it was found that there were significant increases in inflammatory cytokines, IL-1β and IL-6, measured from bronchoalveolar lavage fluid of mice. A single dose Cpl-1 aerosol treatment led to a 4-log reduction in bacterial load in the lungs, prevented bacteremia and sepsis, without significant increases in inflammatory cytokine levels in lavage fluid of Cpl-1 treated mice. Delivery via aerosol is in clinical use for other biologics so the translational readiness would be relatively high, provided that the stability and dosing of endolysins can be established.

#### 2.1.2 Nebulization

The use of commercial jet or mesh nebulizers has been studied using native Cpl-1 and a chimeric endolysin, ClyJ (Wang et al., 2020). Although ClyJ lost activity with either jet or mesh nebulization, mesh nebulization did not change the activity in Cpl-1 under any condition and only prolonged jet nebulization led to conformational changes of the enzyme. Mesh nebulized Cpl-1 at doses of 39.1–50.2 μg/mL showed a significant log reduction in *S. pneumoniae in vitro* when compared to the negative control without endolysin treatment, and a similar log reduction when compared to non-nebulized Cpl-1. Nebulizers are FDA-approved medical devices, although compatibility would need to be validated for each endolysin individually.

#### 2.1.3 Dry powder formulation

A dry powder formulation of Cpl-1 was shown to be effective while also having better stability at room temperature, storage capabilities, and transportation compared to liquid formulations required for nebulization (Wang et al., 2023). Similar to the nebulization studies, Cpl-1 maintained its activity against *S. pneumoniae* after drying into powder form. Regulatory precedent for inhaled drugs exists for powder formulations, however the inhalation of proteins in powder form needs to be further evaluated for particle deposition, immunogenicity, and long-term stability.

#### 2.1.4 Nanotechnology-based delivery

The use of nanotechnology approaches to stabilize endolysins has shown recent success. In general, nanotechnology in drug delivery systems utilizes nanostructures and/or nanoparticles to deliver drugs targeted to specific areas in a controlled manner (Patra et al., 2018). As an example, the SET-M33 peptide, a non-natural antimicrobial peptide synthesized in branched form, was electrostatically attached to dextran nanoparticles and shown to be efficacious *in vivo* against *Pseudomonas aeruginosa* in the lungs (Falciani et al., 2020). Adapting a similar nanosystem to be more specific to *S. pneumoniae*, for instance by incorporating an endolysin, could be a promising strategy. In the case of Cpl-1, nanoparticle delivery systems have already been explored, specifically through the use of chitosan nanoparticles to evaluate effects on Cpl-1 bioavailability (Gondil et al., 2020). Chitosan nanoparticles were chosen given their biodegradability, low immunogenicity, low toxicity, and the fact that they are already well-studied as a delivery platform (Ragelle et al., 2014; Mohammed et al., 2017). At 1 mg/mL, the highest concentration tested, *in vitro* epithelial cell viability after 24 h of incubation was 78 ± 2.2% for Cpl-1-loaded chitosan nanoparticles compared to 79 ± 3.6% for chitosan nanoparticles alone. This study also demonstrated the ability of chitosan nanoparticles to slowly release Cpl-1, thus marking it as a possible candidate for *in vivo* testing against *S. pneumoniae* (Gondil et al., 2020). Currently, nanoparticle systems for endolysin delivery remain preclinical and would require safety and biodistribution studies, in addition to further *in vivo* efficacy validation.

Collectively, the studies described above have shown the efficacy of Cpl-1 to treat *S. pneumoniae* through inhalation, nebulization, powder formulation, and nanosystem delivery, although the use of these technologies for other endolysins active against *S. pneumoniae* remains to be defined. Perhaps better alternatives exist through combinatorial solutions, such as using both nanoparticle technology and dry powder formulation (Sabuj et al., 2021). Multiple methods have been developed for drug delivery to the lungs that have positively impacted treatment outcomes; however, there remains a need for further testing of different endolysins within these delivery systems to fully explore treatment effectiveness. Continued innovation in delivery methods will be critical to realizing the therapeutic potential of endolysins for treating pneumococcal pulmonary infections.

## 3 Anatomical niche: middle ear hurdles and solutions

The *S. pneumoniae* from the nasopharynx reaches the ear via the Eustachian tube (ET) where it establishes infection leading to otitis media (OM) (Loughran et al., 2019). Nasopharyngeal viral infections worsen the condition by activating cytokines, which increase inflammation, thereby aiding *S. pneumoniae* adherence and invasion. Constriction of the ET due to inflammation generates negative pressure, allowing *S. pneumoniae* to ascend into the middle ear to cause disease (Silva, 2022). OM is one of the most common bacterial infections in children and a leading cause of pediatrician appointments and antibiotic prescriptions (Rovers et al., 2004). The short, wide structure of children’s ET makes them more vulnerable to OM (Silva, 2022). Age, alongside genetic influences and environmental factors, work together with sibling presence and daycare attendance to contribute to OM pathogenesis (Rovers, 2008). Acute OM is characterized by effusion in the middle ear paired with one or more of the following symptoms: otalgia, otorrhea, fever, or irritability. OM patients who receive antibiotic treatment may still face recurrent infections that can trigger permanent hearing loss including cholesteatoma development. Surgical procedures including tympanostomy tube placement and adenoidectomy serve as alternative treatments to antibiotics and prophylactic vaccination (Qureishi et al., 2014).

The tympanic membrane (TM) is the tissue separating the outer ear from the middle ear, preventing bacteria, water, and other outside elements from entering the deeper parts of the ear (Kim et al., 2024). The TM stands as a significant barrier to treatment, as passage through it would be required for direct drug targeting of middle ear infections. While oral antibiotics are often prescribed for OM, the limited vasculature of the middle ear often reduces their effectiveness, signifying a need for treatment methods that offer a more direct route. This raises the critical question: how can one effectively pass the barrier between the outer and middle ear to enable direct drug targeting?

Endolysins that can externally cross the tympanic membrane would enable more direct and efficient treatment of middle ear infections such as OM. Recent studies have identified specific peptides capable of crossing the tympanic membrane. These peptides were first discovered using phage display technology, and through continued studies, three peptides demonstrated the most success *in vivo* in rat models of OM (Kurabi et al., 2018a). Prior to optimization from 12-mer to 18-mer peptides, one specific peptide, TM3, was demonstrated to be able to cross an intact human TM discarded during otologic surgery, providing validation in an *ex vivo* model (Kurabi et al., 2018b). Moreover, these peptides were shown to be able to transport large cargos (e.g., whole phage) across the TM, as well as smaller molecules, such as covalently bonded amoxicillin or neomycin (Kurabi et al., 2025). Unfortunately, covalently linking chemical antibiotics to the peptides resulted in a loss of their antimicrobial activity. However, following a similar concept, endolysins active against *S. pneumoniae* could be engineered onto these trans-TM peptides to enable penetration of the membrane for therapeutic delivery. Other groups have also utilized these peptides for delivery into the TM to treat OM; however, rather than delivering antibiotics, they used the peptides to transport V_2_O_5_ nanowires (Liu et al., 2024). The nanowires were able to successfully penetrate the TM, further demonstrating the potential of these peptides to deliver a variety of therapeutic cargoes across the TM for treatment.

Another avenue to explore in terms of engineering endolysins to improve accessibility to the middle ear would be the use of hydrogels for treatment. In the context of wound infections, hydrogels loaded with the endolysin LysP53 against *Acinetobacter baumannii* were able to kill the pathogen and promote wound healing; similar results were observed with the endolysin ClyC against *S. aureus* (Yang et al., 2025). The combination of a peptide-endolysin fusion loaded onto a hydrogel could be a promising strategy for treating OM due to its ability to maintain proximity and release to the TM. Sustained release is particularly important because, as stated above, limited vascularization in the middle ear restricts the effectiveness of systemically delivered antibiotics. In addition, repeated topical dosing can cause irritation, which lowers compliance (Patel et al., 2019). Notably, the use of hydrogel-based delivery vehicles has already been shown to be effective *in vivo* in other ear disease models, including animal models of OM, where they have been shown to prolong local exposure to drugs and enhance their therapeutic effects (Bruk et al., 2020). Additional work has also been conducted utilizing the *S. pneumoniae* endolysin MSlys loaded onto a PEGylated liposome (Silva et al., 2022). *Ex vivo* studies using human TMs showed the PEGylated liposomes improved transport by 6- to 7-fold compared to free endolysin at both 2 h and 24-h time points. Further, the liposomes retained MSlys anti-pneumococcal activity at 2 h post-transport. PEGylated liposomes are commonly used for systemic protein and peptide delivery *in vivo*, however, their use for middle ear delivery has only been tested in *ex vivo* models. Overall, these studies demonstrate the promise of engineering endolysin-based strategies that can overcome the anatomical barrier of the TM and enable more effective treatment of OM.

## 4 Anatomical niche: invasive/systemic infection hurdles and solutions

The third niche involves scenarios where *S. pneumoniae* can migrate into the bloodstream and cause systemic infections, such as bacteremia or sepsis. Once *S. pneumoniae* enters the bloodstream, it must evade innate immune defenses to survive and disseminate to secondary sites. The ability of *S. pneumoniae* to persist in the blood is maintained by several virulence factors, including the polysaccharide capsule, pneumolysin, and surface proteins that inhibit complement activation. When the infection becomes severe, *S. pneumoniae* crosses the blood–brain barrier (BBB), ultimately reaching the cerebrospinal fluid (CSF) and causing meningitis. This translocation of the BBB occurs either through tissue damage or via internalization of *S. pneumoniae* within recruited leukocytes at the membrane (Marra and Brigham, 2001). Within the CSF, *S. pneumoniae* triggers a strong inflammatory response, ultimately leading to neuronal damage and high mortality rates if left untreated.

With respect to bloodstream infections, previous studies have demonstrated that endolysins can provide protection in a bacteremia model when administered via intraperitoneal injection. One specific study combined Cpl-1 and Pal to treat mice challenged with 5 × 10^7^ CFU of *S. pneumoniae*. A single dose of 200 μg of either endolysin administered 1 h post-infection protected mice and reduced bacteremia (Jado et al., 2003). Moreover, as previously noted in this same manuscript, synergistic effects were observed with the combination of Cpl-1 and Pal. However, treatment via the bloodstream raises important questions regarding both half-life and immunogenicity.

Regarding half-life, Cpl-1 is known to form dimers in the presence of choline, and stabilization of the Cpl-1 dimer was shown to increase its half-life by ten-fold in murine plasma (Resch et al., 2011). However, this effect has not been studied in other endolysins, and not all endolysins possess the ability to dimerize. In another approach, it was found that fusing LysK, a *S. aureus*-targeting endolysin, to an albumin-binding domain increased the half-life of LysK by 48% and decreased kidney and liver deposition. However, the bacteriolytic activity of this chimera was only ~18% of the unmodified LysK (Seijsing et al., 2018). Lastly, recent studies have utilized fatty acid derivatization technology to create an endolysin mutant with increased half-life and decreased clearance rates in the blood. In this work, endolysin LysECD7 was fused with lipopolysaccharide-interacting peptides and a valine to cysteine mutation allowed for conjugation with a C16 fatty acid side chain via reaction with acetyl bromide (Li et al., 2024). *In vitro* and *in vivo* studies testing efficacy and pharmacokinetics of this variant against *A. baumannii* showed that the mutant was removed from plasma at a 2.5-fold lower rate and exhibited a 3.9-fold increase in half-life compared to the unmodified enzyme.

Immunogenicity is another potential hurdle for systemic use of endolysins. Computational methods, such as the EpiSweep program, have been used to remove T cell epitopes of lysostaphin, an endolysin-like cell wall hydrolase that targets *S. aureus*, allowing lysostaphin to evade immune recognition (Blazanovic et al., 2015). Furthermore, additional studies on lysostaphin demonstrated greater treatment efficacy with T cell epitope-optimized variants compared to the wild-type enzyme (Zhao et al., 2015). Additionally, the EndoScan technology, a high-throughput linear B cell epitope mapping approach, was recently used to identify immunogenic regions in the *S. pneumoniae*-specific Cpl-1 and Pal endolysins (Harhala et al., 2023). Using this method, amino acids contributing to antibody binding were pinpointed, and engineered Pal variants were created. One Pal variant (280–282: DKP → GGA) demonstrated significantly enhanced antibacterial activity while successfully evading neutralization by antibodies raised against the wild-type enzyme. An overview of representative approaches to enhance the pharmacokinetics of endolysins for systemic administration, such as dimerization, fusion with albumin-binding domain, fatty acid derivatization, and removal of T cell epitopes, is summarized in Table 3.

**Table 3:** Strategies to improve pharmacokinetics of endolysins for systemic use.

Strategy	Example endolysin(s)	Outcome	Reference
Dimer stabilization	Cpl-1	Stabilized dimer formation decreased plasma clearance ~10-fold in mice; increased anti-pneumococcal activity	Resch et al. (2011)
Albumin-binding domain fusion	LysK	Increased half-life by 48%; reduced kidney and liver deposition; activity decreased to ~18% of unmodified enzyme	Seijsing et al. (2018)
Fatty acid derivatization	LysECD7	3.9X increase in half-life; 2.5X slower plasma clearance in mice	Li et al. (2024)
T cell epitope removal	Lysostaphin (endolysin-like)	Reduced immune recognition; enhanced efficacy *in vivo*	Blazanovic et al. (2015) and Zhao et al. (2015)

For *S. pneumoniae* meningitis, therapeutic treatment faces the hurdle of crossing the BBB. The BBB is critical for protecting the central nervous system from pathogens and other harmful agents, but its structure and selectivity make it difficult for treatments to penetrate (Jiao et al., 2024). This challenge is underscored by the study by Valente *et al*., which showed that the endolysin PlyAZ3a was unable to cross the BBB in pharmacokinetic experiments using an infant rat model of *S. pneumoniae* meningitis (Valente et al., 2022). Notably, intracisternal injection of Cpl-1 against *S. pneumoniae* meningitis in a similar infant rat model demonstrated efficacy, decreasing *S. pneumoniae* counts by three logs with a single injection of 20 mg/kg, suggesting that if transit of the BBB can be achieved, *S. pneumoniae* endolysins are likely to be effective in the CSF.

Currently, several physical, biological, and chemical methods are being explored to optimize drug delivery across the BBB, including nanoparticle-based delivery systems (Jiao et al., 2024). Much like the strategies described for crossing the tympanic membrane, cell-penetrating peptides (CPPs) have been utilized to facilitate crossing of the BBB. Peptides such as TAT and xB3 have been discovered to cross the membrane in other therapeutic contexts (Bolhassani et al., 2017; Eyford et al., 2021) and could similarly be applied to deliver endolysins. To date, this application has only been proposed theoretically for endolysins, however, it is worth noting that TAT and other CPPs have been tested as fusions with endolysins for intracellular delivery into epithelial cells, where they demonstrated successful cellular uptake (Becker et al., 2016). Additionally, research into the use of exosomes as antimicrobial delivery systems has been expanding (Jiao et al., 2024). This “Trojan horse” approach could also be adapted for endolysins by exploiting tissue with low immunogenicity to enable entry into the central nervous system. Although promising, the application of CPP- and exosome-based strategies for delivery of endolysins across the BBB remains to be experimentally validated *in vivo*.

## 5 Conclusion

Research has demonstrated the effectiveness of endolysins against *S. pneumoniae*, but significant hurdles remain that must be overcome to achieve optimal therapeutic efficacy. This review explored specific challenges within the context of the major niches of infection: pulmonary, middle ear, and invasive/systemic. All niches present accessibility issues for endolysin treatment, whether due to limited delivery to the lungs, the need to cross the TM to reach the middle ear, or the formidable obstacle of the BBB. Within the pulmonary niche, work has been conducted on aerosolization, nebulization, and powder formulations for Cpl-1, with opportunities for future research into additional endolysins and nanosystem delivery methods. The middle ear and invasive/systemic niches, by contrast, remain unexplored areas, particularly in the context of endolysin-peptide fusions. The success of endolysins to combat *S. pneumoniae* infections relies on developing engineered adaptations that overcome the distinct barriers of different anatomical areas.

Developing new delivery methods remains essential for maximizing the therapeutic potential of endolysins as targeted treatments. To translate these promising agents into clinical practice, rigorous pharmacokinetic, immunogenicity, and toxicity studies are urgently needed, alongside well-designed efficacy trials in relevant preclinical models and eventual human studies. As most applications of endolysins and engineered variants will be classified as biologics, early consideration of regulatory pathways, including requirements for manufacturing quality, safety of novel excipients, and precedent from other endolysin clinical programs, will be key to optimize development and approval.
